# An Enhanced Grey Wolf Optimization Based Feature Selection Wrapped Kernel Extreme Learning Machine for Medical Diagnosis

**DOI:** 10.1155/2017/9512741

**Published:** 2017-01-26

**Authors:** Qiang Li, Huiling Chen, Hui Huang, Xuehua Zhao, ZhenNao Cai, Changfei Tong, Wenbin Liu, Xin Tian

**Affiliations:** ^1^College of Physics and Electronic Information Engineering, Wenzhou University, Wenzhou 325035, China; ^2^School of Digital Media, Shenzhen Institute of Information Technology, Shenzhen 518172, China; ^3^Cancer Hospital, Chinese Academy of Medical Sciences and Shenzhen Hospital, Shenzhen 518000, China

## Abstract

In this study, a new predictive framework is proposed by integrating an improved grey wolf optimization (IGWO) and kernel extreme learning machine (KELM), termed as IGWO-KELM, for medical diagnosis. The proposed IGWO feature selection approach is used for the purpose of finding the optimal feature subset for medical data. In the proposed approach, genetic algorithm (GA) was firstly adopted to generate the diversified initial positions, and then grey wolf optimization (GWO) was used to update the current positions of population in the discrete searching space, thus getting the optimal feature subset for the better classification purpose based on KELM. The proposed approach is compared against the original GA and GWO on the two common disease diagnosis problems in terms of a set of performance metrics, including classification accuracy, sensitivity, specificity, precision, *G*-mean, *F*-measure, and the size of selected features. The simulation results have proven the superiority of the proposed method over the other two competitive counterparts.

## 1. Introduction

In order to make the best medical decisions, medical diagnosis plays a very important role for medical institutions. As everyone knows, false medical diagnoses will lead to incorrect medical decisions, which are likely to cause delays in medical treatment or even loss of patients' life. Recently, a number of computer aided models have been proposed for diagnosing different kinds of diseases, such as diagnostic models for Parkinson's disease [1, 2], breast cancer [3, 4], heart disease [5, 6], and Alzheimer's disease [7, 8]. As a matter of fact, medical diagnosis could be treated as a problem of classification. In the medical diagnosis field, datasets usually contain a large number of features. For example, colorectal microarray dataset [9] contains two thousand features with highest minimal intensity across sixty-two samples. However, there are irrelevant/redundant features in dataset which may reduce the classification accuracy. Feature selection is proposed to solve this problem. The process of a typical feature selection method consists of four basic steps [10]: (1) generation: generate the candidate subset; (2) evaluation: evaluate the subset; (3) stopping criterion: decide when to stop; (4) validation: check whether the subset is valid. Based on whether the evaluation step includes a learning algorithm or not, feature selection methods can be classified into two categories: filter approaches and wrapper approaches. Filter approaches are independent of any learning algorithm and often computationally less expensive and more general than wrapper approaches, while wrapper approaches evaluate the feature subsets with a learning algorithm and usually produce better results than filter approaches for particular problems.

In medical diagnosis scenario, high diagnostic performance is always preferred, even a slight lift in accuracy can make significant difference. Therefore, the wrapper approach is adopted to obtain the better classification performance in this study. Generally, metaheuristics are commonly used for finding the optimal feature subset in wrapper approaches. As a vital member of metaheuristics family, evolutionary computation (EC) has attracted great attention. Many EC based methods in the literature have been proposed to perform feature selection. Raymer et al. [11] suggested using genetic algorithms (GA) to select features. Authors in [12–14] proposed to use binary particle swarm optimization (PSO) for feature selection. Zhang and Sun applied tabu search in feature selection [15]. Compared with above-mentioned EC techniques, grey wolf optimization (GWO) is a new EC technique proposed recently [16]. GWO mimics the social hierarchy and hunting behavior of grey wolves in nature. Due to its excellent search capacity, it has been successfully applied to many real-world problems since its introduction, like optimal reactive power dispatch problem [17], parameter estimation in surface waves [18], static VAR compensator controller design [19], blackout risk prevention in a smart grid [20], capacitated vehicle routing problem [21], nonconvex economic load dispatch problem [22], and so on. However, it should be noted that the initial population of original GWO is generated in a random way. It may result in the lack of diversity for the wolf swarms during the search space. Many studies [23–26] have shown that the quality of the initial population may have a big impact on the global convergence speed and the quality of final solution for the swarm intelligence optimization algorithms, and initial population with good diversity is very helpful to improve the performance of optimization algorithms. Motivated by this core idea, we made the first attempt to use GA to generate a much more appropriate initial population, and then a binary version of GWO was constructed to perform the feature selection task based on the diversified population. On the other hand, to find the most discriminative features in terms of classification accuracies, the choice of an effective and efficient classifier is also of significant importance. In this study, the kernel extreme learning machine (KELM) classifier is adopted to evaluate the fitness value. The KELM is selected due to the fact that it can achieve comparative or better performance with much easier implementation and faster training speed in many classification tasks [27–29].

The main contributions of this paper are summarized as follows:

- A novel predictive framework based on an improved grey wolf optimization (IGWO) and KELM method is presented.
- GA is introduced into the IGWO to generate the more suitable initial positions for GWO.
- The developed framework, IGWO-KELM, is successfully applied to medical diagnosis problems and has achieved superior classification performance to the other competitive counterparts.

The remainder of this paper is organized as follows.  Section 2 gives some brief background knowledge of KELM, GWO, and GA. The detailed implementation of the IGWO-KELM method will be explained in Section 3.  Section 4 describes the experimental design in detail. The experimental results and discussions of the proposed approach are presented in Section 5. Finally, the conclusions are summarized in Section 6.

## 2. Background

### 2.1. Kernel Extreme Learning Machine (KELM)

The traditional back propagation (BP) learning algorithm is a stochastic gradient least mean square algorithm. The gradient of each iteration is greatly affected by the noise interference in the sample. Therefore, it is necessary to use the batch method to average the gradient of multiple samples to get the valuation of the gradient. However, in the case of a large number of training samples, this method is bound to increase the computational complexity of each iteration, and this average effect will ignore the difference between individual training samples, thereby reducing the sensitivity of learning [30].

KELM is an improved algorithm proposed by Guang-Bin Huang, which combines the kernel function into the original extreme learning machine (ELM) [31]. ELM guarantees the network has good generalization performance, greatly improves the learning speed of the forward neural networks, and avoids many of the problems of gradient descent training methods represented by BP neural networks, like ease of being trapped into local optimum, large iterations, and so on. KELM not only has multidominance of the ELM algorithm, but also combines the kernel function, which nonlinearly maps the linear nonseparable mode to the high-dimensional feature space in order to achieve linear separability and further improve the accuracy rate.

ELM is a training algorithm of single hidden layer feed-forward neural networks (SLFNs). The SLFNs model can be presented as follows [32]:

(1) $$\begin{array}{c} f\left(\;x\;\right)=h\left(\;x\;\right)\beta =H\beta , \end{array}$$

where *x* is sample; *f*(*x*) is the output of neural networks, a class vector in classification; *h*(*x*) or *H* is hidden layer feature mapping matrix; *β* is hidden layer output layer link weight. In the ELM algorithm,

(2) $$\begin{array}{c} \beta =H^{T}{\left(\;HH^{T}+\frac{I}{C}\;\right)}^{-1}T, \end{array}$$

where *T* is a matrix consisting of class flag vectors of the training sample, *I* is unit matrix, and *C* is regularization parameter.

In the case where the hidden layer feature map *h*(*x*) is unknown, the KELM kernel matrix can be defined as follows [33]:

(3) $$\begin{array}{c} \Omega =HH^{T}:\Omega_{i,j}=h\left(\;x_{i}\;\right)\cdot h\left(\;x_{j}\;\right)=K\left(\;x_{i},x_{j}\;\right). \end{array}$$

According to (2) and (3), (1) can be transformed as follows:

(4) fxHβ=HHTHHT+IC−1T=Kx,x1⋮Kx,xNTΩ+IC−1T.

If the radial basis function (RBF) is used as kernel function, also known as Gaussian kernel function [34], which can be defined as follows:

(5) $$\begin{array}{c} K\left(\;x,y\;\right)=\exp\left(\;-\frac{{\left\|x-y\right\|}^{2}}{2\gamma^{2}}\;\right), \end{array}$$

therefore, the regularization parameter *C* and the kernel function parameter *γ* are parameters that need to be tuned carefully. The configuration of *C* and *γ* is an important factor affecting the performance of KELM classifier.

### 2.2. Grey Wolf Optimization (GWO)

**Figure 1:**
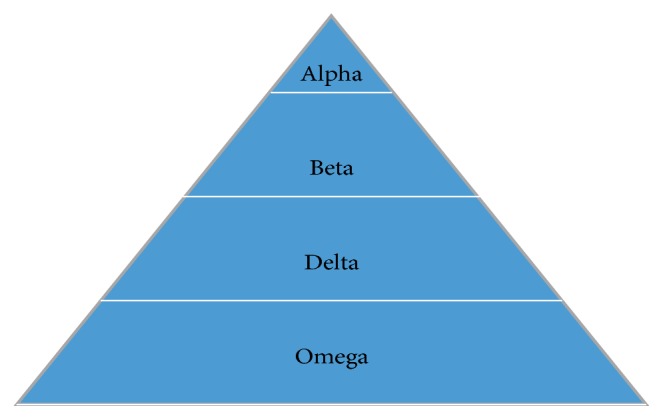
Hierarchy of grey wolves.

The GWO is a new metaheuristic algorithm proposed by Mirjalili et al. [16], which mimics the social hierarchy and hunting mechanism of grey wolves in nature and is based on three main steps: encircling prey, hunting, and attacking prey. In order to mathematically model the leadership hierarchy of wolves, assume the best solution as* alpha*, and the second and third best solutions are named as* beta* and* delta*, respectively. The rest of the candidate solutions are assumed to be* omega*. The strict social dominant hierarchy of grey wolves is shown in Figure 1.

Grey wolves encircle prey during the hunt. In order to mathematically simulate the encircling behavior of grey wolves, the following equations are proposed:

(6) D→=C→·X→preyt−X→wolft,X→wolft+1=X→preyt−A→·D→,

where *t* indicates the current iteration, $\vec{A}$ and $\vec{C}$ are coefficient vectors, ${\vec{X}}_{prey}$ is the position vector of the prey, and ${\vec{X}}_{wolf}$ is the position vector of a grey wolf. The vectors $\vec{A}$ and $\vec{C}$ are calculated as follows:

(7) A→=2a→·r→1−a→,C→=2r→2,

where $\vec{a}$ is linearly decreased from 2 to 0 over the course of iterations and ${\vec{r}}_{1}$ and ${\vec{r}}_{2}$ are random vectors in the interval of [0,1].

**Figure 2:**
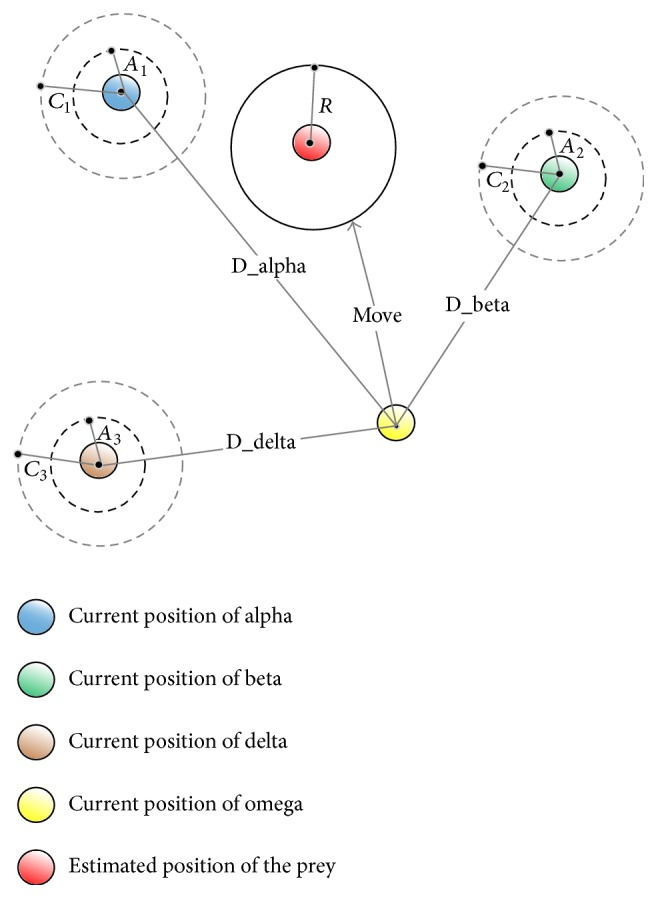
Position updating of grey wolf.

The hunt is usually guided by* alpha*.* Beta* and* delta* might also participate in hunting occasionally. In order to mathematically mimic the hunting behavior of grey wolves, the first three best solutions (*alpha*,* beta,* and* delta*) obtained so far are saved and the other search agents (*omega*) are obliged to update their positions according to (8)–(14). The update of positions for grey wolves is illustrated in Figure 2.

(8) D→alpha=C→1·X→alpha−X→,

(9) D→beta=C→2·X→beta−X→,

(10) D→delta=C→3·X→delta−X→,

(11) X→1=X→alpha−A1→·D→alpha,

(12) X→2=X→beta−A2→·D→beta,

(13) X→3=X→delta−A3→·D→delta,

(14) X→t+1=X→1+X→2+X→33.

The pseudocode of the GWO algorithm is presented as shown in Pseudocode 1.

### 2.3. Genetic Algorithm (GA)

The GA was firstly proposed by Holland [35], which is an adaptive optimization search methodology based on analogy to Darwinian natural selection and genetic in biology systems. In GA, a population is composed of a set of candidate solutions called chromosomes. Each chromosome includes several genes with binary values 0 and 1. In this study, GA was used to generate the initial positions for GWO. The steps of generating initial positions of population by GA are described below.

- *Initialization*. Chromosomes are randomly generated.
- *Selection*. A roulette choosing method is used to select parent chromosomes.
- *Crossover*. A single point crossover method is used to create offspring chromosomes.
- *Mutation*. Uniform mutation is adopted.
- *Decode*. Decode the mutated chromosomes as the initial positions of population.

## 3. The Proposed IGWO-KELM Framework

**Figure 3:**
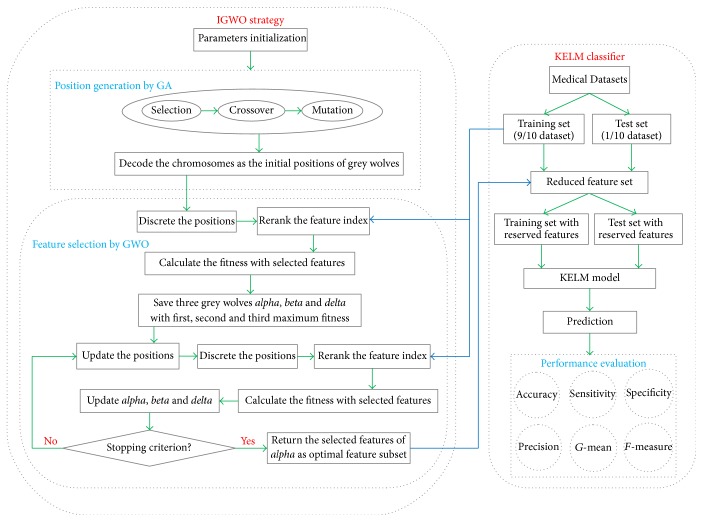
Flowchart of IGWO-KELM.

This study proposed a new computational framework, IGWO-KELM, for medical diagnosis purpose. IGWO-KELM is comprised of two main phases. In the first stage, IGWO is used to filter out the redundant and irrelevant information by adaptively searching for the best feature combination in the medical data. In the proposed IGWO, GA is firstly used to generate the initial positions of population, and then GWO is utilized to update the current positions of population in the discrete searching space. In the second stage, the effective and efficient KELM classifier is conducted based on the optimal feature subset obtained in the first stage.  Figure 3 presents a detailed flowchart of the proposed IGWO-KELM framework.

The IGWO is mainly used to adaptively search the feature space for best feature combination. The best feature combination is the one with maximum classification accuracy and minimum number of selected features. The fitness function used in IGWO to evaluate the selected features is shown as the following equation:

(15) $$\begin{array}{c} Fitness=\alpha P+\beta \frac{N-L}{N}, \end{array}$$

where *P* is the accuracy of the classification model, *L* is the length of selected feature subset, *N* is the total number of features in the dataset, and *α* and *β* are two parameters corresponding to the weight of classification accuracy and feature selection quality, *α* ∈ [0,1] and *β* = 1 − *α*.

**Figure 4:**
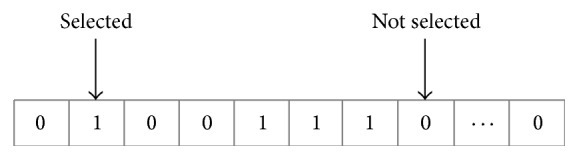
A flag vector for feature selection.

A flag vector for feature selection is shown in Figure 4. The vector consisting of a series of binary values of 0 and 1 represents a subset of features, that is, an actual feature vector, which has been normalized [36]. For a problem with *n* dimensions, there are *n* bits in the vector. The *i*th feature is selected if the value of the *i*th bit equals one; otherwise, this feature will not be selected (*i* = 1,2,…, *n*). The size of a feature subset is the number of bits, whose values are one in the vector. The pseudocode of the IGWO algorithm is presented as shown in pseudocode 2.

## 4. Experimental Design

### 4.1. Data Description

In order to evaluate the proposed IGWO-KELM methodology, two common medical diagnosis problems were investigated, including the Parkinson's disease diagnosis and breast cancer diagnosis. The datasets of Parkinson and Wisconsin diagnostic breast cancer (WDBC) publicly available from the UCI machine learning data repository were used.

**Figure 5:**
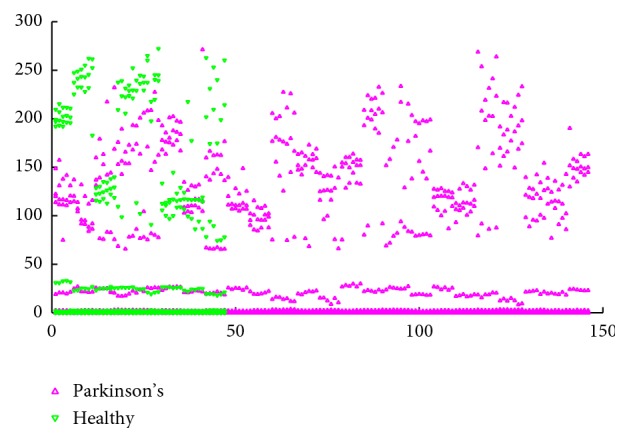
Distribution of the Parkinson dataset.

The Parkinson dataset is composed of a range of biomedical voice measurements from 31 people, 23 with Parkinson's disease (PD). Each column in the table is a particular voice measure, and each row corresponds to one of 195 voice recordings from these individuals. The main aim of the dataset is to discriminate healthy people from those with PD, given the results of various medical tests carried out on a patient. The time since diagnoses ranged from 0 to 28 years, and the ages of the subjects ranged from 46 to 85 years, with a mean age of 65.8. Each subject provides an average of six phonations of the vowel (yielding 195 samples in total), each 36 seconds in length [37]. The description of Parkinson dataset is presented in Table 1. The Parkinson dataset contains 195 cases, including 147 Parkinson's cases and 48 healthy cases. The distribution of the Parkinson dataset is shown in Figure 5.

The WDBC dataset was created from the University of Wisconsin, Madison, by Dr. Wolberg et al. [38]. The dataset contains 32 attributes (ID, diagnosis, and 30 real-valued input features). Features are computed from a digitized image of a fine needle aspirate (FNA) of a breast mass. They describe the characteristics of the cell nuclei presenting in the image. Interactive image processing techniques and linear-programming-based inductive classifier have been used to build a highly accurate system for diagnosing breast cancer. With an interactive interface, the user initializes active contour models, known as snakes, near the boundaries of a set of cell nuclei. The customized snakes are deformed to the exact shape of the nuclei. This allows for precise automated analysis of nuclear size shape and texture. Ten such features are computed for each nucleus and the mean value largest (or “worst”) value and standard error of each feature are found over the range of isolated cells [39], and they are described as follows.

*Descriptions of Features of the WDBC Dataset*

- *Radius*. The mean of distances from center to points on the perimeter
- *Texture*. The standard deviation of grey-scale values
- *Perimeter*. The total distance between consecutive snake points
- *Area*. The number of pixels on the interior of the snake adds one-half of the pixels on the perimeter
- *Smoothness*. The local variation in radius lengths
- *Compactness*. Perimeter^2^/area - 1.0
- *Concavity*. The severity of concave portions of the contour
- *Concave Points*. The number of concave portions of the contour
- *Symmetry*. The length difference between lines perpendicular to the major axis to the nuclear boundary in both directions
- *Fractal Dimension*. “Coastline approximation” - 1

**Figure 6:**
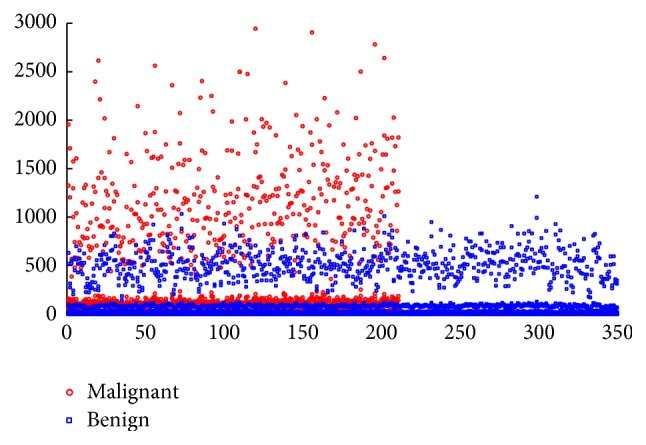
Distribution of the WDBC dataset.

The mean value, worst (mean of the three largest values), and standard error of each feature were computed for each image, resulting in a total of thirty features for each case in the dataset. There are 569 samples' data out of which 357 samples are labeled as benign breast cancer and the remaining as malignant breast cancer patients. The distribution of the WDBC dataset is shown in Figure 6.

### 4.2. Experimental Setup

The experiments were conducted in the MATLAB platform, which ran on Windows 7 ultimate operating system with Intel® Core™ i3-3217U CPU (1.80 GHz) and 8 GB of RAM. The implementation of KELM by Huang is available at http://www3.ntu.edu.sg/home/egbhuang. The IGWO, GWO, and GA were implemented from scratch.

In this study, the data were scaled into [−1,1] by normalization for the facility of computation. In order to acquire unbiased classification results, the* k*-fold cross validation (CV) was used [40]. This study took 10-fold CV to test the performance of the proposed algorithm. However, only one time of running the 10-fold CV will result in the inaccurate evaluation. So the 10-fold CV will run ten times.

Regarding the parameter choice of KELM, different penalty parameters *C* = {2^−5^, 2^−4^,…, 2^4^, 2^5^} and different kernel parameters *γ* = {2^−5^, 2^−4^,…, 2^4^, 2^5^} were taken to find the best classification results. In other words, 11 × 11 = 121 combinations were tried for each method. The final experimental results demonstrate that when *C* is equal to 2^5^ (32) and *γ* is equal to 2^−1^ (0.5), KELM achieves the best performance. Therefore, *C* and *γ* for KELM are set to 32 and 0.5 in this study, respectively. The global and algorithm-specific parameter setting is outlined in Table 2.

### 4.3. Performance Evaluation

Considering a two-class classifier, formally, each instance is mapped to one element of the set {*P*, *N*} of positive and negative class labels. A classifier is a mapping from instances to predicted classes and produces a discrete class label indicating only the predicted class of the instance. A confusion matrix contains information about actual and predicted classifications done by a classification system. Performance of such systems is commonly evaluated using the data in the matrix as shown in Table 3.

Once the model has been built, it can be applied to a test set to predict the class labels of previously unseen data. It is often useful to measure the performance of the model with test data, because such a measure provides an unbiased estimate of generation errors. In this study, we evaluate the prediction models, utilizing the KELM classifier, based on different evaluation criteria described below.

Accuracy is the proportion of the total number of predictions that were correct. It is determined using

(16) $$\begin{array}{c} Accuracy=\frac{TP+TN}{TP+FP+FN+TN}\times 100\% \end{array}$$

Sensitivity is the proportion of positive instances that were correctly classified, as calculated using

(17) $$\begin{array}{c} Sensitivity=\frac{TP}{TP+FN}\times 100\% \end{array}$$

Specificity is the proportion of negative instances that were correctly classified, as calculated using

(18) $$\begin{array}{c} Specificity=\frac{TN}{TN+FP}\times 100\% \end{array}$$

Precision is the proportion of the predicted positive instances that were correct, as calculated using

(19) $$\begin{array}{c} Precision=\frac{TP}{TP+FP}\times 100\% \end{array}$$

The accuracy determined using (16) may not be an adequate performance measure when the number of negative instances is much greater than the number of positive instances. Other performance measures account for this by including sensitivity in literature. For example, Kubat and Matwin [41] proposed the geometric mean (*G-*mean) metric in 1998, as defined using

(20) $$\begin{array}{c} G\text{-mean}=\sqrt{Sensitivity*Specificity}. \end{array}$$

Lewis and Gale [42] proposed the* F-*measure metric in 1994, as defined using

(21) $$\begin{array}{c} F\text{-measure}=\frac{\left(\beta^{2}+1\right)*Precision*Sensitivity}{\beta^{2}*Precision+Sensitivity}. \end{array}$$

In (21), *β* has a value from 0 to infinity and is used to control the weight assigned to precision and sensitivity. Any classifier evaluated using (21) will have a measure value of 0, if all positive instances are classified incorrectly. The value of *β* is set to 1 in this study.

## 5. Experimental Results and Discussions

Comparative experiments were performed between IGWO-KELM and the other two competitive methods, including GWO-KELM and GA-KELM, in order to evaluate the effectiveness of the proposed method for the two disease prediction problems. 10-fold CV was used to estimate the classification results of each approach; the mean values over ten times of 10-fold CV were taken as the final experiment results.

### 5.1. Parkinson's Disease Prediction

**Figure 7:**
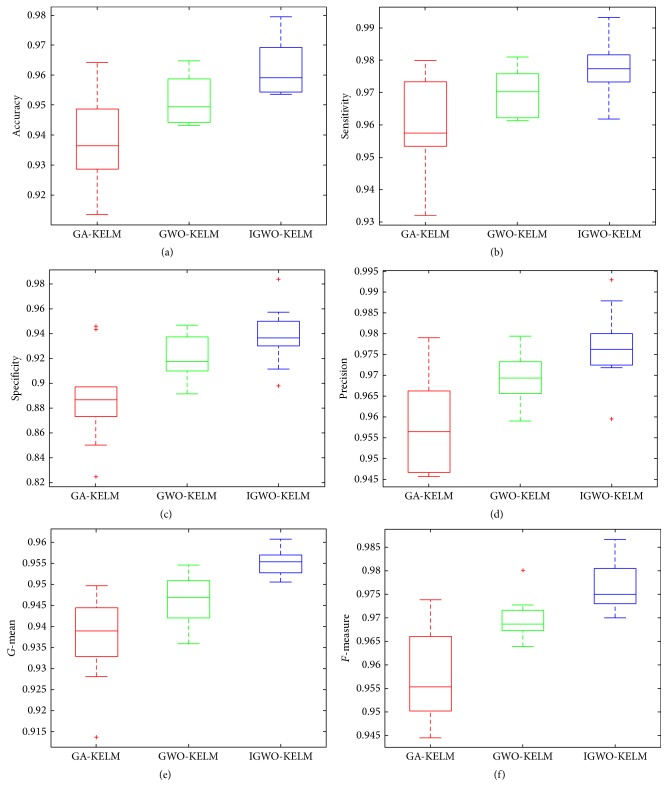
Box plots for 10 times of trials for each classification method on the Parkinson dataset.

Table 4 illustrates the detailed classification results of the three methods in terms of the number of selected features, accuracy, sensitivity, specificity, precision,* G*-mean, and* F*-measure on the Parkinson dataset. It can be seen in Table 4 that, among the three methods, the IGWO-KELM method performs the best with the least number of selected features, with the highest values of 97.45% accuracy, 98.08% sensitivity, 96.67% specificity, 99.29% precision, 97.37%* G-*mean, and 98.68%* F-*measure and with the smallest standard deviation as well. The box plots in Figure 7 graphically depict comparisons among IGWO-KELM versus the other two methods in terms of accuracy, sensitivity, specificity, precision,* G-*mean, and* F-*measure. IGWO-KELM displays the greatest performance among the three methods. Specially, for the measurement specificity, as shown in Figure 7(c), the median value obtained from IGWO-SVM is 93.83%, much higher than GA-KELM and GWO-KELM by 88.74% and 91.99%, respectively.

**Figure 8:**
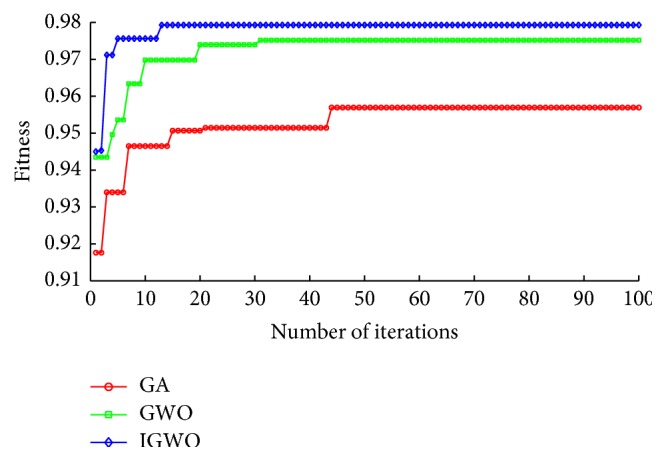
Fitness comparison among three algorithms on the Parkinson dataset.

To observe the optimization procedure of the algorithms including GA, GWO, and IGWO, the iteration process was recorded in Figure 8. It can be seen from Figure 8 that the fitness curve of IGWO completely converges after the 17th iteration, while the fitness curves of GWO and GA completely converge after the 30th iteration and the 45th iteration, respectively. It indicates that the proposed IGWO is much more effective than the other two methods and can quickly find the best solution in the search space. Moreover, we can also observe that the fitness value of IGWO is always bigger than that of GWO and GA in the whole iteration course.

The population size and the iteration number are two key factors in swarm intelligence algorithms; thus their suitable values were investigated on the Parkinson dataset. Firstly, to find the best value of the population size, different sizes of population from 4 to 20 with the step of 4 were taken when the number of iterations was fixed to 100. It can be observed from Table 5 that the performance of IGWO-KELM is shown to be the best when the iteration number is equal to 8. Secondly, to find the best value of the iteration number, the size of population was fixed to 8 and different numbers of iterations from 50 to 250 with step of 50 were tried. As shown in Table 6, IGWO-KELM achieves the best performance when the iteration number is equal to 100. Therefore, to obtain the best performance of the proposed method for the Parkinson dataset, the size of population and the number of iterations were set to 8 and 100, respectively, in this study.

**Figure 9:**
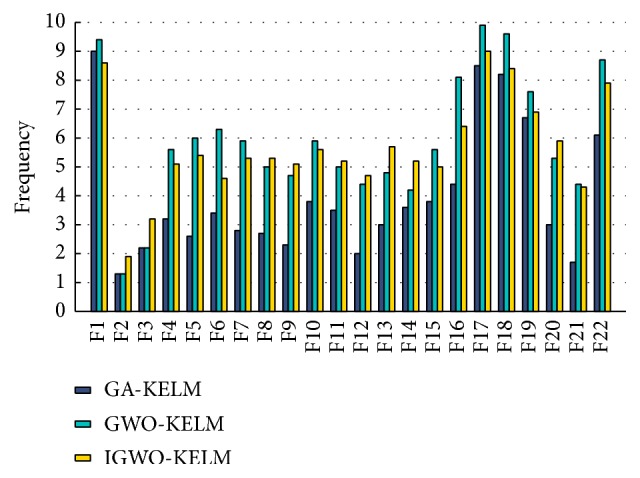
Frequency comparisons among three methods for each feature of the Parkinson dataset.

Figure 9 shows the selected frequency of each feature of the Parkinson dataset in the process of the feature selection by three methods, including GA-KELM, GWO-KELM, and IGWO-KELM. It can be found from Figure 9 that the frequencies of the 8th feature, the 9th feature, the 11th feature, the 13th feature, the 14th feature, and the 20th feature selected by IGWO-KELM are higher than the counterparts selected by GA-KELM and GWO-KELM, and the frequency of these features selected by IGWO-KELM is more than five. It indicates that the 8th feature, the 9th feature, the 11th feature, the 13th feature, the 14th feature, and the 20th feature are much more important features than others in the Parkinson dataset.

Table 7 presents the average selected times of features of the Parkinson dataset, ranging from 1 to 10. Firstly, for IGWO-KELM, the average selected times of the 1st feature, the 4th feature, the 5th feature, the 8th feature, the 9th feature, the 10th feature, the 11th feature, the 13th feature, the 14th feature, the 16th feature, the 17th feature, the 18th feature, the 19th feature, the 20th feature, and the 22nd feature are more than five times. Secondly, for GWO-KELM, the average selected times of the 1st feature, the 4th feature, the 5th feature, the 6th feature, the 7th feature, the 10th feature, the 15th feature, the 16th feature, the 17th feature, the 18th feature, the 19th feature, the 20th feature, and the 22nd feature are more than five times. Thirdly, for GA-KELM, the average selected times of the 1st feature, the 17th feature, the 18th feature, the 19th feature, and the 22nd feature are more than five times. It is interesting to find that the average selected times of five features including the 1st feature (MDVP: Fo), the 17th feature (RPDE), the 18th feature (D2), the 19th feature (DFA), and the 22nd feature (PPE) are all more than five times for IGWO-KELM, GWO-KELM, and GA-KELM. It indicates that the three methods are highly consistent to pick out the most important features for the Parkinson dataset. It also suggests that these features should be paid more attention to in the decision-making process.

### 5.2. Brest Cancer Prediction

**Figure 10:**
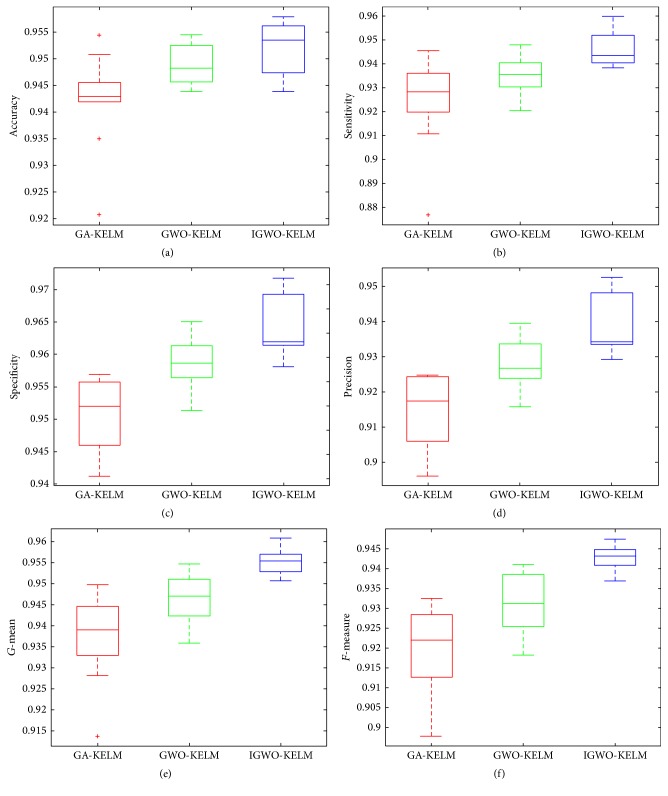
Box plots for 10 times of trials for each classification method on the WDBC dataset.

Table 8 presents the detailed classification results of the three methods in terms of the number of selected features, accuracy, sensitivity, specificity, precision,* G-*mean, and* F*-measure on the WDBC dataset. From the table, it can be seen that the IGWO-KELM method achieves the highest performance among the three methods with results of 95.78% accuracy, 94.88% Sensitivity, 96.75% Specificity, 95.24% Precision, 95.81%* G-*mean, and 95.06%* F-*measure. The boxplots are drawn to exhibit the general values of the accuracy, sensitivity, specificity, precision,* G-*mean, and* F-*measure and they are shown in Figure 10. As expected, compared with the other two methods, IGWO-KELM yields consistent increase of all performance measurements. For example, for the measurement sensitivity, as can be observed in Figure 10(b), the median value obtained from IGWO-SVM is 94.62%, higher than GA-KELM and GWO-KELM by 92.44% and 93.52%, respectively.

**Figure 11:**
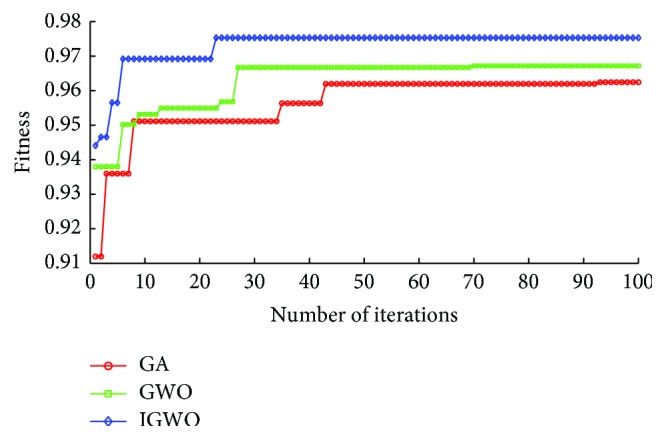
Fitness comparison among three algorithms on the WDBC dataset.

Figure 11 shows the optimization procedure of the algorithms including GA, GWO, and IGWO. It can be observed from Figure 11 that the fitness curve of IGWO completely converges after the 24th iteration, while the fitness curve of GWO and GA just started to converge from the 28th iteration and the 43rd iteration, respectively. Moreover, it can also be observed that the fitness value of IGWO is always bigger than that of GWO or GA in the whole iteration course. It indicates that IGWO not only converges more quickly, but also obtains better solution quality than GA and GWO. The main reason may lie in that the GA initialization helps GWO to search more effectively in search space; thus outperforming both GA and GWO in converging to a better result.

As done for the Parkinson dataset, the population size and the iteration number were also investigated on the WDBC dataset. Firstly, to find the best value of the population size, different sizes of population from 4 to 20 with the step of 4 were taken when the number of iterations was fixed to 100. It can be observed from Table 9 that the performance of IGWO-KELM is shown to be the best when the iteration number is equal to 8. Secondly, to find the best value of the iteration number, the size of population was fixed to 8 and different numbers of iterations from 50 to 250 with step of 50 were tried. As shown in Table 10, IGWO-KELM achieves the best performance when the iteration number is equal to 100. Therefore, to obtain the best performance of the proposed method for the WDBC dataset, the size of population, and the number of iterations were set to 8 and 100, respectively, in this study.

**Figure 12:**
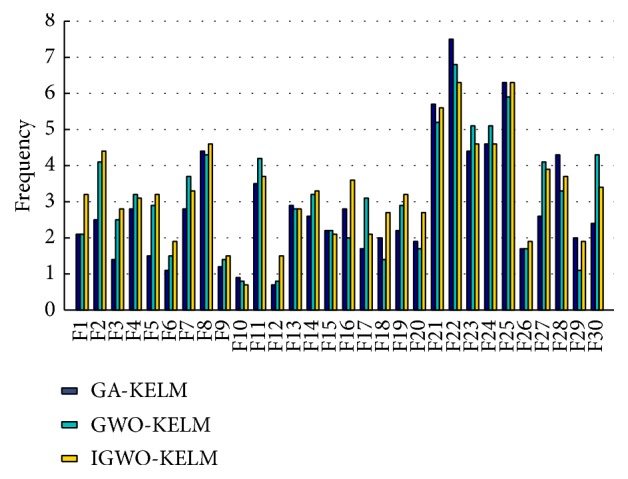
Frequency comparisons among three methods for each feature of the WDBC dataset.

Figure 12 shows the selected frequency of each feature of the WDBC dataset in the course of the feature selection by GA-KELM, GWO-KELM, and IGWO-KELM. It can be observed from Figure 12 that the frequencies of the 1st feature, the 2nd feature, the 3rd feature, the 5th feature, the 6th feature, the 8th feature, the 9th feature, the 12th feature, the 14th feature, the 16th feature, the 18th feature, the 19th feature, the 20th feature, and the 26th feature selected by IGWO-KELM are higher than the counterparts selected by GA-KELM and GWO-KELM. It indicates that these chosen features are important features in the WDBC dataset; they should be paid more attention to when the doctors make a decision.

Table 11 presents the average selected times of features of the WDBC dataset, ranging between 1 and 10. On the one hand, to IGWO-KELM and GA-KELM, the average selected times of the 21st feature, the 22nd feature, and the 25th feature are more than five times. On the other hand, to GWO-KELM, the average selected times of the 21st feature, the 22nd feature, the 23rd feature, the 24th feature, and the 25th feature are more than five times. Therefore, it can be deduced that the 21st feature, the 22nd feature, and the 25th feature are important features in the WDBC dataset, since they are selected consistently by the three methods in a high frequency.

## 6. Conclusions

In this paper, an IGWO-KELM methodology is described in detail. The proposed framework consists of two main stages which are feature selection and classification, respectively. Firstly, an improved grey wolf optimization, IGWO, was proposed for selecting the most informative features in the specific medical data. Secondly, the effective KELM classifier was used to perform the prediction based on the representative feature subset obtained in the first stage. The proposed method is compared against well-known feature selection methods including GA and GWO on the two disease diagnosis problems using a set of criteria to assess different aspects of the proposed framework. The simulation results have demonstrated that the proposed IGWO method not only adaptively converges more quickly, producing much better solution quality, but also gains less number of selected features, achieving high classification performance. In future works, we will apply the proposed methodology to more practical problems and plan to implement our method in a parallel way with the aid of high performance tools.

## Figures and Tables

**Pseudocode 1:**
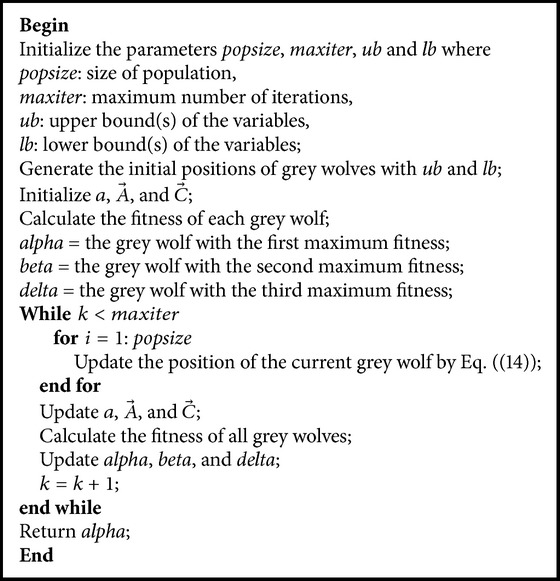
Pseudocode of the GWO algorithm.

**Pseudocode 2:**
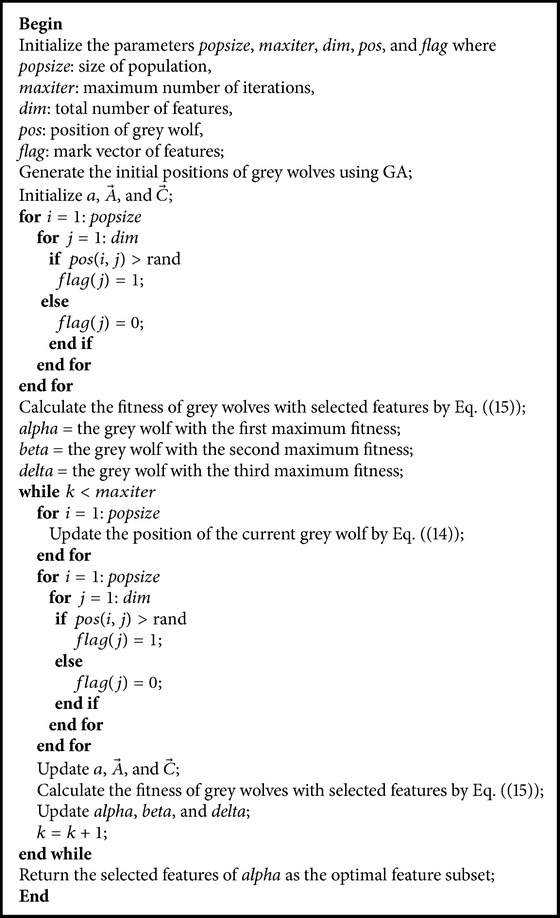
Pseudocode of the IGWO algorithm.

**Table 1:** Descriptions of attributes of the Parkinson dataset.

	Attribute	Description
F1	MDVP: Fo (Hz)	Average vocal fundamental frequency

F2	MDVP: Fhi (Hz)	Maximum vocal fundamental frequency

F3	MDVP: Flo (Hz)	Minimum vocal fundamental frequency

F4	MDVP: Jitter (%)	Several measures of variation in fundamental frequency
F5	MDVP: Jitter (Abs)	
F6	MDVP: RAP	
F7	MDVP: PPQ	
F8	Jitter: DDP	

F9	MDVP: Shimmer	Several measures of variation in amplitude
F10	MDVP: Shimmer (dB)	
F11	Shimmer: APQ3	
F12	Shimmer: APQ5	
F13	MDVP: APQ	
F14	Shimmer: DDA	

F15	NHR	Two measures of ratio of noise to tonal components in the voice
F16	HNR	

F17	RPDE	Two nonlinear dynamical complexity measures
F18	D2	

F19	DFA	Signal fractal scaling exponent

F20	Spread1	Three nonlinear measures of fundamental frequency variation
F21	Spread2	
F22	PPE	

**Table 2:** Parameter setting for experiments.

Parameter	Value(s)
*K* for cross validation	10
Size of population	8
Number of iterations	100
Problem dimension	*n* ^*∗*^
Search domain	[0,1]
Crossover probability in GA	0.8
Mutation probability in GA	0.01
*α* in the fitness function	0.99
*β* in the fitness function	0.01
*C* for KELM	32
*γ* for KELM	0.5

^*∗*^ *n* is the total number of features.

**Table 3:** Confusion matrix.

		Actual class	
		*P*	*N*
Predicted class	*P*	True positive (TP)	False positive (FP)
	*N*	False negative (FN)	True negative (TN)

TP: the number of correct predictions that an instance is positive.

FP: the number of incorrect predictions that an instance is positive.

FN: the number of incorrect predictions that an instance is negative.

TN: the number of correct predictions that an instance is negative.

**Table 4:** Experimental results of three methods on the Parkinson dataset.

Method	Features' size	Accuracy (%)	Sensitivity (%)	Specificity (%)	Precision (%)	*G*-mean (%)	*F*-measure (%)
IGWO-KELM	9.2 ± 2.01	97.45 ± 2.65	98.08 ± 2.11	96.67 ± 5.27	99.29 ± 2.26	97.37 ± 3.13	98.68 ± 1.78
GWO-KELM	10.7 ± 2.16	95.37 ± 3.55	97.90 ± 2.64	94.62 ± 8.64	98.00 ± 3.22	96.29 ± 4.96	97.99 ± 2.22
GA-KELM	9.4 ± 2.96	94.89 ± 3.70	96.30 ± 3.09	92.49 ± 9.09	97.95 ± 3.30	94.39 ± 5.35	97.13 ± 2.40

**Table 5:** Experimental results of IGWO-KELM with different population size on the Parkinson dataset.

Population size (iteration number = 100)	Accuracy (%)	Sensitivity (%)	Specificity (%)	Precision (%)	*G*-mean (%)	*F*-measure (%)
4	94.34	96.71	87.67	95.95	92.08	96.33
**8**	**96.97**	**98.16**	**94.99**	**97.99**	**96.57**	**98.08**
12	93.37	96.04	88.48	95.29	92.18	95.66
16	95.39	96.75	92.67	97.29	94.69	97.16
20	93.24	94.70	88.83	96.62	91.72	95.65

**Table 6:** Experimental results of IGWO-KELM with different iteration number on the Parkinson dataset.

Iteration number (population size = 8)	Accuracy (%)	Sensitivity (%)	Specificity (%)	Precision (%)	*G*-mean (%)	*F*-measure (%)
50	95.89	97.99	91.48	96.62	94.68	97.30
**100**	**97.45**	**99.38**	**93.48**	**97.33**	**96.38**	**98.34**
150	95.92	97.33	93.81	97.29	95.55	97.31
200	95.50	98.08	91.89	95.99	94.94	97.03
250	95.92	97.33	92.67	97.29	94.97	97.31

**Table 7:** Average selected times of features by three methods on the Parkinson dataset.

Feature	Average selected times		
	GA- KELM	GWO- KELM	IGWO- KELM
F1	9	9.4	8.6
F2	1.3	1.3	1.9
F3	2.2	2.2	3.2
F4	3.2	5.6	5.1
F5	2.6	6	5.4
F6	3.4	6.3	4.6
F7	2.8	5.9	5.3
F8	2.7	5	5.3
F9	2.3	4.7	5.1
F10	3.8	5.9	5.6
F11	3.5	5	5.2
F12	2	4.4	4.7
F13	3	4.8	5.7
F14	3.6	4.2	5.2
F15	3.8	5.6	5
F16	4.4	8.1	6.4
F17	8.5	9.9	9
F18	8.2	9.6	8.4
F19	6.7	7.6	6.9
F20	3	5.3	5.9
F21	1.7	4.4	4.3
F22	6.1	8.7	7.9

**Table 8:** Experimental results of three methods on the WDBC dataset.

Method	Features' size	Accuracy (%)	Sensitivity (%)	Specificity (%)	Precision (%)	*G*-mean (%)	*F*-measure (%)
IGWO-KELM	8.7 ± 2.74	95.7 ± 1.43	94.88 ± 3.51	96.75 ± 2.57	95.24 ± 3.35	95.81 ± 1.65	95.06 ± 1.94
GWO-KELM	9.8 ± 2.58	94.9 ± 1.89	93.34 ± 4.10	95.38 ± 2.66	94.87 ± 4.56	94.35 ± 2.16	94.10 ± 2.78
GA-KELM	9.4 ± 3.07	93.4 ± 2.19	92.91 ± 4.19	94.13 ± 2.69	94.81 ± 4.58	93.52 ± 2.84	93.85 ± 2.79

**Table 9:** Experimental results of IGWO-KELM with different population size on the WDBC dataset.

Population size (iteration number = 100)	Accuracy (%)	Sensitivity (%)	Specificity (%)	Precision (%)	*G*-mean (%)	*F*-measure (%)
4	95.08	94.63	95.68	92.45	92.15	93.53
**8**	**95.61**	**95.73**	**96.09**	**92.95**	**95.91**	**94.32**
12	94.56	93.41	95.37	92.04	94.39	92.72
16	94.38	92.29	95.72	92.45	93.99	92.37
20	95.26	94.52	95.97	92.90	95.24	93.70

**Table 10:** Experimental results of IGWO-KELM with different iteration number on the WDBC dataset.

Iteration number (population size = 8)	Accuracy (%)	Sensitivity (%)	Specificity (%)	Precision (%)	*G*-mean (%)	*F*-measure (%)
50	93.67	92.83	94.58	90.54	93.70	91.67
**100**	**95.43**	**94.01**	**96.65**	**94.33**	**95.32**	**94.17**
150	95.25	93.25	95.64	93.85	94.44	93.55
200	94.90	94.01	96.44	92.45	95.22	93.22
250	93.15	89.99	95.27	92.01	92.59	90.99

**Table 11:** Average selected times of features by three methods on the WDBC dataset.

Feature	Average selected times		
	GA- KELM	GWO- KELM	IGWO- KELM
F1	2.1	2.1	3.2
F2	2.5	4.1	4.4
F3	1.4	2.5	2.8
F4	2.8	3.2	3.1
F5	1.5	2.9	3.2
F6	1.1	1.5	1.9
F7	2.8	3.7	3.3
F8	4.4	4.3	4.6
F9	1.2	1.4	1.5
F10	0.9	0.8	0.7
F11	3.5	4.2	3.7
F12	0.7	0.8	1.5
F13	2.9	2.8	2.8
F14	2.6	3.2	3.3
F15	2.2	2.2	2.1
F16	2.8	2	3.6
F17	1.7	3.1	2.1
F18	2	1.4	2.7
F19	2.2	2.9	3.2
F20	1.9	1.7	2.7
F21	5.7	5.2	5.6
F22	7.5	6.8	6.3
F23	4.4	5.1	4.6
F24	4.6	5.1	4.6
F25	6.3	5.9	6.3
F26	1.7	1.7	1.9
F27	2.6	4.1	3.9
F28	4.3	3.3	3.7
F29	2	1.1	1.9
F30	2.4	4.3	3.4
